# Copper Nanoparticles for Printed Electronics: Routes Towards Achieving Oxidation Stability

**DOI:** 10.3390/ma3094626

**Published:** 2010-09-08

**Authors:** Shlomo Magdassi, Michael Grouchko, Alexander Kamyshny

**Affiliations:** Casali Institute of Applied Chemistry, the Institute of Chemistry, the Hebrew University of Jerusalem, Jerusalem 91904, Israel; E-Mail: magdassi@cc.huji.ac.il

**Keywords:** printed electronics, copper nanoparticles, conductive inks

## Abstract

In the past few years, the synthesis of Cu nanoparticles has attracted much attention because of its huge potential for replacing expensive nano silver inks utilized in conductive printing. A major problem in utilizing these copper nanoparticles is their inherent tendency to oxidize in ambient conditions. Recently, there have been several reports presenting various approaches which demonstrate that copper nanoparticles can resist oxidation under ambient conditions, if they are coated by a proper protective layer. This layer may consist of an organic polymer, alkene chains, amorphous carbon or graphenes, or inorganic materials such as silica, or an inert metal. Such coated copper nanoparticles enable achieving high conductivities by direct printing of conductive patterns. These approaches open new possibilities in printed electronics, for example by using copper based inkjet inks to form various devices such as solar cells, Radio Frequency Identification (RFID) tags, and electroluminescence devices. This paper provides a review on the synthesis of copper nanoparticles, mainly by wet chemistry routes, and their utilization in printed electronics.

## 1. Introduction

The use of silver nanoparticles (NPs) in conductive inks and their printing by inkjet technology has been known for years [1]. However, the very high cost of silver limits wide industrial application. Since copper is much cheaper, but possesses a very high conductivity (only 6% less than that of Ag), Cu NPs can be considered as a replacement for silver NPs.

Therefore, during recent years, the synthesis of Cu NPs has become of great interest from a scientific as well as an industrial point of view, due to its huge potential for replacing the expensive nano silver ink. Since the 1990s, many attempts have been made to synthesize nano copper by wet chemistry, as well as by gas or solid phase methods. Among these, are the sonochemical method [2], microemulsion techniques [3,4,5], polyol processes [6,7,8], radiation methods [9,10,11,12,13], thermal reduction [14], reducing flame synthesis [15,16], metal vapor synthesis [17], vacuum vapor deposition [18,19] and chemical reduction in solution [20,21]. An example of the gas phase synthesis route is the pioneering work of Luechinger *et al*. [16], in which various metal nanoparticles were prepared by the flame-aerosol route, which is based on spraying a metal precursor yielding metal nanoparticles coated by a carbon layer.

The present review focuses mainly on wet chemical processes which are based on the reduction of metal ions by reducing agents in liquid media. This route is very convenient since it may result in various dispersions with controllable particle characteristics, by numerous variable experimental parameters. The main parameters are the type and concentration of reagents, their redox potentials and rate of addition, type and concentration of protective agents, temperature, pH and the addition of preformed seeds [22]. Additional advantages of these processes are that the metallic NPs can be synthesized with essentially low-cost equipment and in large volumes.

The driving force of the ion metal reduction (mOx^n+^ + Red = mOx^0^ + Red^mn+^) is the difference in the redox potentials of the reagents (∆E) which correlates with the Gibb's free energy of the reaction at standard conditions:

∆G^0^ = −RTlnK = −nF∆E^0^

(where n = the number of electrons in a reaction equation, K = equilibrium constant, F = Farady’s constant).

The reduction is thermodynamically possible only if the redox potential of the reducer is more negative than that of the oxidizer (metal precursor) and, respectively, ΔE is positive. This difference should be larger than 0.3–0.4 V, otherwise the reduction will proceed too slowly and may not result in the formation of NPs [23]. Therefore, the synthesis of stable copper NPs presents an additional challenge in relation to gold and silver, which is caused by a relatively low Cu^0^/Cu^2+^ redox potential (+0.34 V).

As presented in Table 1, not too many papers have been published thus far on the various ways of synthesizing copper NPs by chemical reduction in solution.

**Table 1:** A list of published methods to synthesize copper NPs by chemical reduction in solution.

#	Solvent	Cu precursor	Reducer	Stabilizer	Particle size	Ref.
1	Water	CuSO_4_	Sodium borohydrate	SDS	2–10 nm	Lisiecki 1996 [24]
2	Water + n-hexanol/cyclohexane	CuCl_2_	Sodium borohydrate	TX-100	5–15 nm	Qi 1997 [5]
3	Water+ n-Heptane, n-Octane, n-Hexane	CuCl_2_	Sodium borohydrate	HDEHP	40–80 nm	Song 2004 [25]
4	Isooctane	Cu(AOT)_2_	Hydrazine	Na AOT	10–30 nm	Salzemann 2004 [26]
5	Water	CuCl_2_	Hydrazine	CTAB	5 nm	Wu 2004 [27]
6	Ethylene Glycol	CuSO_4_	Ascorbic acid	PVP 40	100 nm (cubes)	Wang 2006 [28]
7	Water	Cu(NO_3_)_2_	Ascorbic acid	PVP 58	3 nm	Wu 2006 [29]
8	Di-Ethylene Glycol	CuSO_4_	Sodium phosphinate	PVP 40	45 nm	Park 2007 [7]
9	Octyl ether	Cu(acac)_2_	1,2-hexadecanediol	Oleic acid, oleyl amine	5–200 nm	Mott 2007 [30]
10	Toluene + water	CuCl_2_	Sodium borohydrate	Lauric acid + TOAB	3 nm	Kanninen 2008 [31]
11	Di-Ethylene Glycol	CuCl_2_	SFS	PVP	50 nm	Khanna 2009 [32]
12	Water	Cu(NO_3_)_2_	Hydrazine	PAA Na	20–100 nm	Grouchko 2009 [21]

SFS = sodium formaldehyde sulfoxylate; HDEHP = Bis(ethylhexyl)hydrogen phosphate; SDS = Sodium dodecyl sulfate; Na AOT = Sodium bis(2-ethylhexyl) sulfosuccinate; CTAB = Cetyl trimethylammonium bromide; PVP = Polyvinylpyrrolidone; TOAB = Tetraoctylammonium bromide; PAA Na = Polyacrylic acid sodium salt.

However, a major problem in utilizing these copper NPs is their inherent tendency to oxidize in ambient conditions [33]. Yanase *et al*. [34,35] examined the mechanism and kinetics of oxidation and reduction of copper NPs in oxygen and hydrogen atmospheres by using UV-vis spectroscopy. They found a fast oxidation process from Cu to CuO_0.67_ and a slow one from CuO_0.67_ to CuO, confirming the formerly suggested mechanism reported by Wieder *et al*. [36]. From the point of view of conductive printing applications, the presence of copper oxides on the surface of NPs, has two negative consequences: it increases the required sintering temperature, and reduces the electrical conductivity.

Only a limited number of reports have attempted to address the oxidation problem, which in general is based on minimizing the exposure of the copper NPs to oxygen, by a protective layer composed of a second material at the surface of the particles.

These materials can be divided into four groups: (1) carbon-based materials (carbon and graphene), (2) surfactants and polymers, (3) silica, (4) metals.

In this paper we describe the approaches that are meant to achieve air stable copper NPs.

## 2. Amorphous Carbon and Graphene Based Materials

Li *et al*. [37] synthesized copper NPs by means of solid state reduction of cupric carbonate by glucose at high temperature. The fundamental reactions can be summarized as follows:

$$\begin{array}{l} CuCO_{3}\cdot Cu{\left(OH\right)}_{2}\cdot xH_{2}O\overset{350^{o}C}{\to }2CuO+CO_{2}+(x+1)H_{2}O\\ C_{6}H_{12}O_{6}\cdot H_{2}O\overset{350^{o}C}{\to }6C+7H_{2}O\\ CuO+C\overset{350^{o}C}{\to }Cu+CO \end{array}$$

It was found that as the glucose load increases, the carbon layer on the surface of the NPs becomes thicker, and stable copper NPs, coated by amorphous carbon, are obtained.

**Figure 1:**
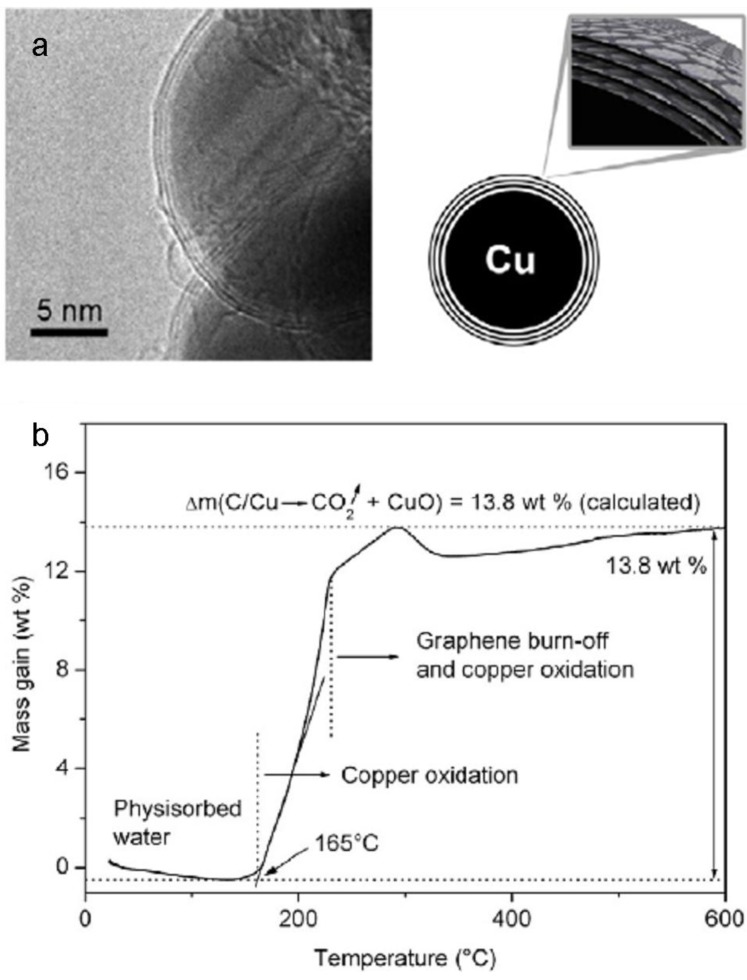
(**a**) Transmission electron microscope (TEM) image of a single copper NP with a thick graphene layer of 3 nm (left) and a corresponding schematic illustration (right); (**b**) Thermogravimetric analysis (TGA) confirms the thermal stability of these NPs up to 165 °C. Reprinted from reference [16], with permission from IOP Publishing LTD.

Luechinger *et al*. [16] has demonstrated how deposition of bi- or tri-layers graphene on copper can be realized on a large scale, and how it enables full protection of the copper metal core under humid air. The protected copper was synthesized by the reducing flame technique [15,38] which leads to the formation of ~50 nm copper NPs coated with a ~3 nm layer of graphene. The main advantage of this method is that it leads to coated nanoparticles in a one-step process. Figure 1a presents a high resolution transmission electron microscope (HR-TEM) image of copper NPs coated by graphene, and a corresponding schematic illustration. It was found that the graphene-coated copper NPs are stable to oxidation up to 165 °C (thermogravimetric analysis, Figure 1b). The formulation of these NPs in an aqueous ink-jet ink and printing with that ink enabled conductive patterns. As a highly conductive material, graphene is not expected to decrease the pattern conductivity, even though, the obtained conductivity was found to be five orders of magnitude lower than that of bulk copper.

## 3. Surfactants and Polymers

Another way to address this oxidation problem is to coat the obtained copper NPs with a dense layer of capping agents. Such molecules are usually present at the surface of the NPs to prevent aggregation and agglomeration in dispersions. However, in order to minimize the penetration of oxygen to the NPs surface, a dense structure of these molecules is required.

Ang *et al*. [39] formed 5 to 10 nm Cu NPs coated by alkanethiols, by reducing the copper nitrate in presence of C_8_ to C_12_ alkanethiols in ethanol. In a typical procedure copper nitrate was dissolved in absolute alcohol and the respective alkanethiol was added. Then, a solution of sodium borohydride was added dropwise into the reaction mixture. The reaction was maintained at room temperature with vigorous stirring carried out in an inert N_2_ atmosphere. A brownish precipitate was isolated by centrifugation, washed repeatedly with deionized water, toluene, ethanol, and acetone, and vacuum dried [39]. It was found that such a layer is a good barrier that protects the copper NPs from oxidation for at least six months. Kannienen *et al*. [31] used the same approach with a wider range of chain lengths (C_6_ to C_18_), and compared their performance to that of lauric (C_12_) and oleic (C_18_) acids. Thiols were found to improve oxidation resistance already at a 1:1 ratio, while oxidation resistance improved with increased chain length, but not with increased concentration. Oleic acid was found to increase oxidation resistance slightly in comparison to lauric acid. In summary, it was found that oleic acid-capped particles were clearly superior to thiol-stabilized particles.

**Figure 2:**
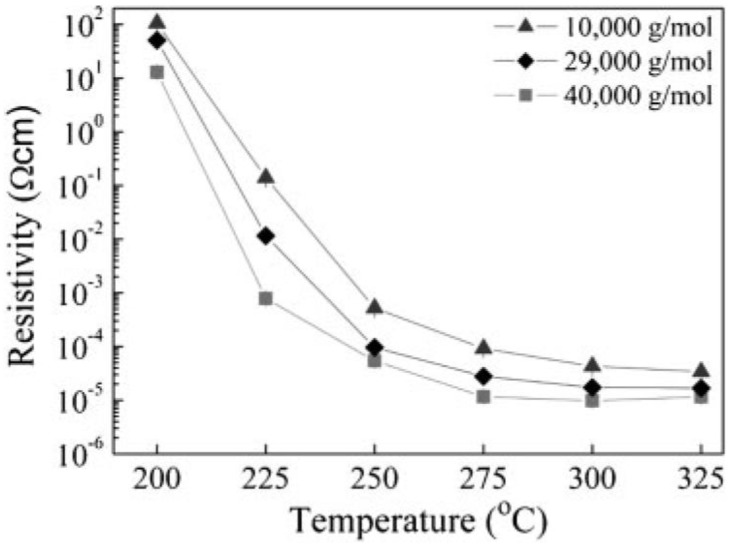
Resistivity of the Cu conductive film as a function of heat treatment temperature for various PVP MWs. The ink-jet printed Cu nanoparticulate films were heat-treated at various temperatures between 200 °C and 325 °C under vacuum. Reprinted from [40], Reproduced with permission of copyright Wiley-VCH Verlag GmbH and Co. KGaA.

More stable dispersions were obtained by the use of polymers as capping agents. For example, Jeong *et al*. [40] followed by Engels *et al*. [41] evaluated the effect of the well established stabilizer, poly(N-vinylpyrrolidone) (PVP), at various molecular weights (10,000, 29,000 and 40,000 g/mol). Jeong *et al*. found that besides the effect of the PVP molecular weight on the NPs size (the particle size increases as the PVP molecular weight increases), the minimum thickness of the amorphous CuO and chemisorbed PVP layers was 1.6 nm, obtained using PVP with a molecular weight of 40000 g/mol. The molecular weight of the PVP capping agent determines their conformation when adsorbed on the Cu surface, which significantly influences the formation of the surface oxide layer. In addition, their results confirm that the thickness of the surface oxide layer is the predominant factor which determines the electrical conductivity of the Cu film obtained after sintering. As presented in Figure 2, when the molecular weight of PVP increases, the obtained resistivity decreases for all temperature ranges down to six times the resistivity of bulk copper (under vacuum). Lee *et al*. [8] used the same polymer, PVP 40, to form a conductive copper ink, and to ink-jet print with it to form conductive patterns. According to TGA analysis, the obtained copper patterns were stable to oxidation up to 180 °C. However, highly conductive patterns (45% of bulk copper) were demonstrated only after heating to 200 °C for 60 min under hydrogen. Kobayashi *et al*. [42] used polypyrrole (PPY), a conductive polymer, to coat the copper NPs through the polymerization of pyrrole in aqueous solution. This method produced PPY-coated copper NPs which were chemically stable, even under air, for a prolonged period of time. However, no data was shown regarding the stability of these NPs at elevated temperatures. Pulkkinen *et al*. [43] evaluated two different polymers, polyethylene imine (PEI) and tetraethylenepentamine (TEPA), as protecting agents for copper NPs. However, even after pressing and heating to 250 °C, the obtained resistivities were 3 to 5 orders of magnitude higher than bulk copper.

## 4. Silica Coating

**Figure 3:**
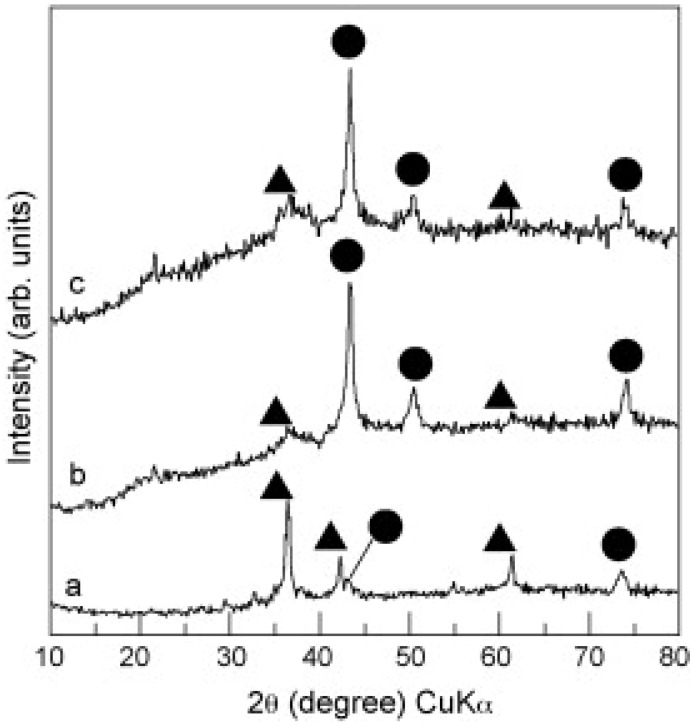
XRD patterns of (**a**) uncoated Cu NPs; (**b**) Cu/SiO_2_ NPs; (**c**) Cu/SiO_2_ NPs one month after preparation. (●) Metallic Cu, (▲) Cu_2_O. Reprinted from reference [44] with permission from Elsevier.

Only one attempt has been made to form an inorganic protection layer on copper NPs. Kobayashi *et al*. [44] reported his success in coating copper NPs by a thin layer of silica. The x-ray diffraction (XRD) analysis in Figure 3 shows that while the uncoated copper NPs are accompanied by clear Cu_2_O peaks (3a), the coated particles have only low Cu_2_O peaks (3b), even after one month (3c). However, in view point of conductive printing, the presence of silica, an insulating material with high melting point, is a major obstacle in obtaining a continuous conductive pattern.

## 5. Metallic Coating

Core-shell structured, bimetallic NPs can be prepared by the successive reduction of one metal over the nuclei of another [45]. The preparation and characterization of bimetallic NPs from various combinations of noble metals have been the subject of numerous papers, examples of which include the Au-Pd [46], Au-Pt [47], Ag-Pd [48], Ag-Pt [49], Ag-Cu [50], and Ag-Au [51] systems. Until now, many methods such as electroless plating [52], surface seeding [53], and self-assembly [54], have been explored to fabricate metallic shells on metallic particles. Generally, such NPs are prepared by the successive reduction of one metal ion over the core of another [55]. This process often leads to the formation of a new nuclei of the second metal in solution (in addition to a shell around the first metal core) and is clearly undesirable from the application point of view. Satry *et al*. [56] overcame this problem in a gold-silver core-shell system by immobilizing a reducing agent on the surface of the core metal which, when exposed to the second metal ions, reduces them, thereby leading to the formation of a thin metallic shell.

Schmid *et al*. [46] used hydroxylamine as a reducing agent that is capable of reducing the metal ion, but the reduction was dramatically accelerated by the Au surfaces. As a result, in the presence of preformed gold NPs, no new particle nucleation occurred in the solution, and all the added metal ions served in the formation of the shell.

**Figure 4:**
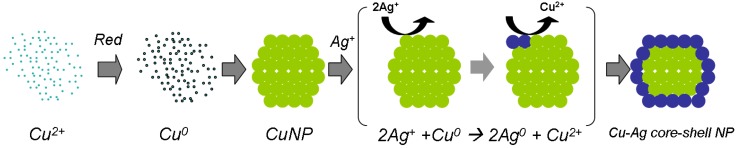
Schematic illustration of a single Cu NP synthesis and the formation of a silver shell by the transmetalation reaction. The surface copper atoms serve as reducing agents for the silver ions.

Furthermore, copper-silver core-shell NPs have been described in several studies, using electrodeposition [57], thermal evaporation techniques under ultrahigh vacuum [50], and ion exchange in soda lime glass matrix [58]. Lee *et al*. [59] demonstrated the formation of various core-shell NPs through the transmetalation (redox) process. It should be noted that one disadvantage of this method is that it requires several steps. In the first step, the core particles are synthesized. Then, the core material serves as a reducer for the reduction of shell atoms on the core particle. The core surface atoms are oxidized (and are released to the solution as ions), and the shell material ions are reduced. In our previous report [60], we used this approach to synthesize a copper-silver core-shell structure in a two step process. As schematically presented in Figure 4; in the first step an aqueous dispersion of Cu NPs is prepared by reducing Cu(NO_3_)_2_ with hydrazine hydrate excess in the presence of polyacrylic acid sodium salt as a polymeric stabilizer [21]. The large excess of hydrazine prevents oxidation of the Cu NPs in the aqueous dispersion, but only if it is kept in closed vials. In the second step, the excess hydrazine is consumed or washed out, and silver salt is added. During the transmetalation reaction, the reduction of silver ions by the copper metal takes place directly on the surface of CuNPs (Ag to Cu atomic ratio of 0.12), thus creating a silver shell on the copper core.

**Figure 5:**
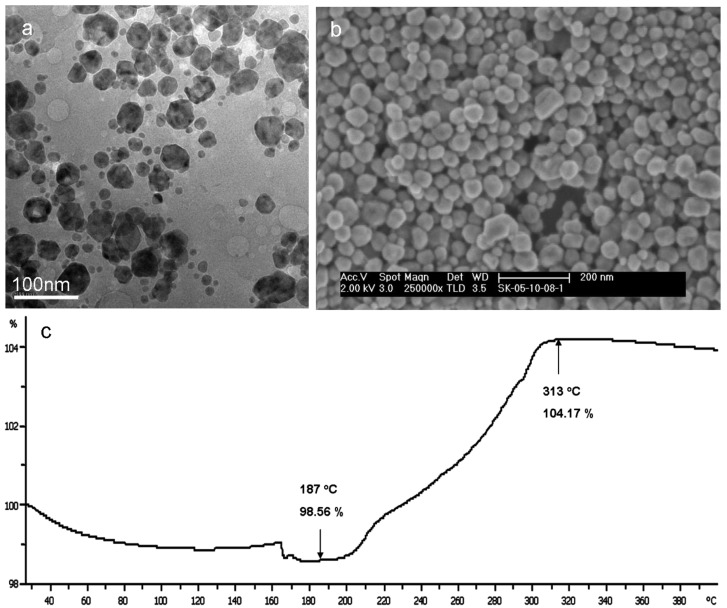
(**a**) TEM and (**b**) SEM images of the obtained copper-silver core-shell NPs; (**c**) TGA of the copper-silver core-shell NPs under air atmosphere.

As follows from the TEM and scanning electron microscopy (SEM) analyses (Figure 5 (a) and (b), respectively), the size distribution of the obtained Cu-Ag core-shell NPs is in the range of 10 to 50 nm. The thermal stability of the obtained NPs was measured by TGA (Figure 5 (c)) confirming the inertness of the copper core up to 187 °C where copper oxides start to appear—leading to a mass increase of about 5 wt %.

**Figure 6:**
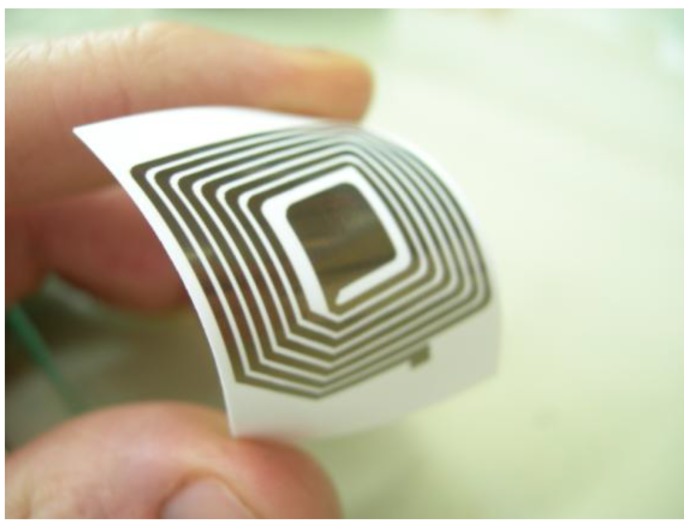
A flexible RFID antenna printed using copper-silver core-shell ink on inkjet photo paper.

By using these oxidation stable Cu NPs, it was possible to further evaluate the use of these NPs in inkjet printing of conductive patterns. An inkjet water-based formulation, which contained 25 wt % Cu-Ag core-shell NPs, was printed by a Lexmark office printer. An example of a Radio Frequency Identification (RFID) antenna, printed on an inkjet photo paper, is presented in Figure 6. However, due to the presence of organic stabilizer at the NPs surface, the resistivity of the obtained patterns was very high. To obtain highly conductive patterns, a sintering process should be carried out. In order to overcome the temperature sensitivity of the substrate (paper, plastics), a new room temperature sintering process was developed [61,62], based on the neutralization of stabilizing charges at the NPs surface. The sheet resistance measurements after the sintering process revealed, as expected, that the sintering is accompanied by a drastic decrease of resistance, down to values of 3 (±0.2) Ω/square. It should be emphasized that such low sheet resistances were reported until now only for metallic patterns which were heated at temperatures of ≥150 °C for prolonged time, while in the present study it was achieved spontaneously at room temperature.

## 6. Summary

In summary, there are only a few reports which present various approaches for obtaining copper nanoparticles that can resist oxidation under ambient conditions. These approaches are based on coating copper NPs by a proper protective layer, thus enabling copper to be used as a low-cost non-noble metal. This layer may consist of an organic polymer, alkane chains, amorphous carbon or graphenes, or inorganic materials such as silica, or an inert metal. Several reports have shown that the formulation of these NPs as conductive inkjet inks, and their printing, can yield conductive patterns which are stable to oxidation for at least several months. Naturally, the formation of a low or non-conductive shell (polymers, surfactants, carbon or silica) on the copper NPs has a negative effect on the obtained conductivity of the printed pattern which is composed of the copper nanoparticles (except for cases in which the shell is graphene or a second metal). This inherent contradiction between conductivity and coating layers is well addressed in the literature. To the best of our knowledge, so far oxidation resistance accompanied by high conductivity was achieved by the formation of a PVP shell [40] or a silver shell [60] on the copper NPs. This opens new possibilities for applications in printed electronics, such as solar cells, RFID tags and electroluminescence (EL) devices.
